# Multiligament Knee Injuries: A 15-Year Clinical and Epidemiological Analysis at a Tertiary Referral Center

**DOI:** 10.7759/cureus.101310

**Published:** 2026-01-11

**Authors:** David Mayorga-Naranjo, Amparo Ortega-Yago, Aranza Pedraza-Corbi, Daniel Bonete-Lluch

**Affiliations:** 1 Department of Orthopedic Surgery and Traumatology, Hospital Universitari i Politècnic La Fe, Valencia, ESP

**Keywords:** anatomical reconstruction, high energy trauma, laprade technique, mlki, multiligament knee injuries, orthopedic surgery

## Abstract

Introduction: Multiligament knee injuries (MLKIs) represent a significant diagnostic and therapeutic challenge and are most often the result of high-energy trauma. The LaPrade technique has become a reproducible option for anatomical reconstruction of the posterolateral corner. This study presents the experience of a tertiary care center in the management of these complex injuries.

Methods: A retrospective descriptive study was conducted on 25 patients with MLKIs treated between 2010 and 2023 at our tertiary care hospital. Demographic and clinical variables, injury type (Schenck classification), mechanism of injury, treatment, and complications were analyzed. Prognostic outcomes were categorized as good or fair-poor based on direct patient-reported assessment at the one-year follow‑up regarding overall functional status, without the use of validated functional scales. Data analysis was performed using R‑Statistics version 3.5.2 (R Foundation for Statistical Computing, Vienna, AUT).

Results: Around 80% of patients were male, with a mean age of 42.1 ± 11.8 years and a mean BMI of 26.8 ± 3.9 kg/m². Traffic accidents were the most common mechanism (56%), followed by sports injuries and falls (20% each). Knee dislocation (KD) V was the most prevalent injury type (40%). Anatomical multiligament reconstruction, primarily following the LaPrade technique, was performed in 44% of cases. Complications occurred in 20% of patients, including joint stiffness, condylar necrosis, and ligament re‑rupture.

Conclusion: Anatomical multiligament reconstruction provides satisfactory functional outcomes with a complication rate comparable to the literature. A multidisciplinary approach and early rehabilitation are essential to optimize functional recovery in complex knee injuries.

## Introduction

Multiligament knee injuries (MLKIs) present a substantial diagnostic and therapeutic challenge due to their complex anatomical involvement, variability in injury patterns, and frequent association with concomitant soft‑tissue or neurovascular injury [1-3]. These lesions typically occur following high‑energy mechanisms such as traffic accidents or falls from height, although they may also arise during high‑demand or contact sports [4].

The true incidence of MLKIs is estimated to be between 0.02% and 0.2% of all knee injuries, though this figure is likely underestimated [5]. The mechanism of injury directly influences the extent of capsuloligamentous damage and the potential for neurovascular compromise, which in turn determines prognosis and guides therapeutic strategy [6,7]. Current international consensus guidelines published by Murray et al. in 2024 recommend surgical management in most cases, particularly in younger patients, with a preference for anatomical ligament reconstruction over repair due to superior functional outcomes and lower failure rates, especially regarding the posterolateral corner (PLC) [8,9]. Staged reconstruction is associated with a reduced risk of arthrofibrosis and improved long‑term outcomes [10].

Management of MLKIs has evolved toward anatomical multiligament reconstruction, prioritizing restoration of knee function and reduction of long‑term complications. The LaPrade technique, which consists of an anatomic reconstruction of the PLC by restoring the lateral collateral ligament, popliteus tendon, and popliteofibular ligament using tendon grafts and anatomical tunnel placement, has become one of the most reproducible and biomechanically validated methods for anatomical PLC reconstruction, demonstrating favorable clinical outcomes and a low rate of failure [11,12].

The primary objective of this retrospective descriptive study is to present 15 years of experience at a tertiary referral center in the management of MLKIs, describing patient epidemiology, mechanisms of injury, injury patterns according to the Schenck classification, treatment strategies, and postoperative complications. Secondary objectives include the descriptive reporting of functional outcomes and the exploratory assessment of their association with injury mechanism and treatment complexity.

## Materials and methods

Study design

A retrospective descriptive study was conducted, including adult patients diagnosed with MLKI of traumatic origin and treated at our tertiary care hospital between October 2010 and October 2023. The study was conducted in accordance with European Good Clinical Practice guidelines and the 2013 Declaration of Helsinki. It was approved by the Institutional Ethics Committee of Institut d’Investigació Sanitària La Fe de València (Valencia, ESP).

Inclusion criteria were age >18 years, traumatic MLKI, and ≥2 years of follow‑up. Exclusion criteria included infection‑related or tumor‑related MLKIs, non‑traumatic ligament injuries, and insufficient follow‑up. Collected variables included sex, age, BMI, laterality, mechanism of injury, Schenck classification type [13], presence of knee dislocation, neurovascular involvement, soft‑tissue compromise, infection, open fracture, and compartment syndrome. Treatment details and clinical evolution were also recorded.

Diagnostic and therapeutic management

The initial assessment included radiographs, CT scans, and MRIs to characterize injury patterns. The MLKIs were categorized using the Schenck classification, distinguishing major ligament involvement as follows: knee dislocation (KD) I (single cruciate rupture), KD II (complete anterior cruciate ligament (ACL) and posterior cruciate ligament (PCL) tears with intact collaterals), KD III (bicruciate injury with injury to either the medial (KD III‑M) or lateral collateral ligament (KD III‑L)), KD IV (injury to both cruciates and both collaterals), and KD V (fracture‑dislocation) [13].

Patients with KD V injuries underwent temporary stabilization with a unilateral external fixator (Hoffmann 3; Stryker, Kalamazoo, MI, USA). Definitive surgery was performed in a single stage in all cases except KD V injuries, which required staged surgical management. The ACL reconstruction utilized anatomical tunnels with interference screw fixation, supplemented with Lemaire lateral extra‑articular augmentation. The PCL reconstruction was performed using double‑bundle arthroscopic techniques with tibial inlay or transtibial approaches and anatomical femoral/tibial fixation. Meniscal tears were repaired arthroscopically. Collateral ligament repair involved anatomical reattachment with augmentation techniques; acute lateral collateral avulsions requiring reinforcement were treated with grafts.

Follow‑up assessments occurred at two weeks, one month, three months, six months, and one year. The final evaluation included an MRI to assess secondary injury and radiographs to detect early gonarthrosis. Recorded complications included stiffness, neurovascular injuries, infection, compartment syndrome, chondropathy, avascular necrosis, and persistent ligamentous or meniscal injury.

Postoperative rehabilitation followed a structured and progressively standardized protocol, with modifications according to the specific ligaments reconstructed. In cases involving ACL reconstruction, early controlled range of motion and progressive weight-bearing were encouraged, while PCL-based reconstructions required more restrictive protocols, including delayed flexion, prone exercises, and prolonged bracing to minimize posterior tibial translation. An extension knee brace was used during the initial postoperative period, typically for the first three weeks, with gradual progression of mobility thereafter. Early rehabilitation goals included pain and edema control, early mobilization within safe limits, and quadriceps activation. Intermediate phases focused on range-of-motion progression, strengthening, and proprioceptive training, with advanced weight-bearing based on the reconstruction type and clinical stability. Final phases emphasized return to functional and sport-specific activities, supported by functional testing and stress radiographs. Although minor refinements were introduced over the 15-year study period, reflecting advances in surgical techniques and rehabilitation principles, the core structure and objectives of the rehabilitation protocol remained consistent, allowing for comparable functional recovery across the cohort.

Functional outcome assessment and statistical analysis

Prognostic outcomes were classified as 'good' or 'fair‑poor' based on a direct question during the one‑year outpatient follow‑up regarding overall functional recovery as perceived by the patient. No validated functional scales (International Knee Documentation Committee (IKDC), Lysholm, Tegner) were used. Data were analyzed using R‑Statistics version 3.5.2 (R Foundation for Statistical Computing, Vienna, AUT). Quantitative variables were summarized as mean, median, and standard deviation. Qualitative variables were expressed as absolute frequencies and percentages.

## Results

Demographic and clinical characteristics

Among 8,743 trauma patients evaluated in our emergency department during the study period, 25 met the inclusion criteria for traumatic MLKI. The cohort included 20 men (80%) and five women (20%), with a mean age of 42.1 ± 11.8 years (median 41). Mean BMI was 26.84 ± 3.87 kg/m² (median 27), placing most patients in the overweight category. Shapiro-Wilk testing confirmed normal distribution for both age (p = 0.88) and BMI (p = 0.66). The left knee was affected in 68% of cases (n = 17). No bilateral cases were observed.

Mechanism of Injury

Traffic accidents were the leading cause of injury (56%), predominantly motorcycle accidents (44%), followed by scooter accidents (12%). Sports injuries accounted for 20% of the cases, mainly football (12%), then skiing and basketball (4% each). Falls also accounted for 20% of cases, including falls from height (8%), falls down stairs (8%), and ground‑level falls (4%). One injury resulted from an accidental twisting mechanism (4%) (Table 1).

**Table 1:** Absolute and relative frequencies of the different mechanisms of MLKIs MLKIs: Multiligament knee injuries

Mechanism of injury	Absolute frequency	Relative frequency (%)
Traffic accident	14	56
Motorcycle	11	44
Scooter	3	12
Sports injury	5	20
Football	3	12
Skiing	1	4
Basketball	1	4
Fall	5	20
Fall from height	2	8
Fall down the stairs	2	8
Ground-level fall	1	4
Knee twisting injury	1	4

Schenck classification and associated injuries

Knee dislocation V was the most common injury pattern (40%), followed by KD I (28%), KD IV (16%), KD III‑L (8%), KD II (4%), and KD III‑M (4%). Knee dislocation occurred in 40% of patients. Neurovascular injury was identified in two cases (8%). Soft‑tissue compromise occurred in 32% (n = 8), including swelling (n = 5) and cutaneous defects (n = 3). One patient (4%) presented with superficial infection. Four patients (20%) had open fractures: three tibial plateau fractures and one combined tibial plateau, medial femoral condyle, and patellar fracture. No compartment syndromes were documented.

Treatment

Ten patients (40%) required temporary external fixation due to severe instability or soft tissue compromise, all of whom sustained high-energy injuries related to traffic accidents. The remaining 15 patients (60%) were initially managed with closed reduction and plaster immobilization.

Time from injury to definite surgical management averaged 2.5 weeks. Staged surgical management was performed exclusively in Schenck V injuries; no other injury patterns required a staged approach. Most patients underwent anatomical multiligament reconstruction combining ACL and/or PCL reconstruction with autografts or allografts using the LaPrade technique (44%). Complex reconstructions requiring additional plating or fixation, indicated for high‑energy unstable patterns or associated fractures, accounted for 20%. Four patients (16%) received selective repairs (ACL, PCL, medial collateral ligament (MCL), or PLC). Three patients (12%) required surgery dedicated to fracture fixation or meniscal injury; when indicated, meniscal repair was performed arthroscopically during the same surgical procedure. One patient (4%) received nonoperative treatment. One patient (4%) died during the perioperative period due to respiratory complications related to the initial high‑energy mechanism (traffic accident) and was not included in the functional outcome analysis.

Functional outcomes and complications

Fifteen patients (62.5%) reported good functional outcomes at the one-year follow-up, while nine patients (37.5%) reported fair-poor outcomes based on their subjective assessment of overall knee function. Several complications were documented during follow-up.

One patient developed significant postoperative stiffness requiring arthrolysis four months after reconstruction. Another patient presented with avascular necrosis of the medial femoral condyle, a rare but serious event likely related to local vascular compromise from the initial trauma or reconstructive procedure. One patient sustained an ACL graft re-rupture. A separate case progressed to advanced chondropathy, consistent with post-traumatic degenerative change. Additionally, one patient demonstrated persistent medial meniscal tearing despite initial repair, ultimately requiring partial meniscectomy and resulting in residual symptoms. No deep infections, compartment syndromes, or neurovascular complications were observed following definitive surgery.

## Discussion

The findings of this series reflect the typical characteristics of MLKIs, with a clear predominance in young male patients, a mean age of 42 years, and high-energy mechanisms, primarily traffic accidents and particularly motorcycle collisions, which accounted for more than half of the cases [1,4,6]. This epidemiological profile aligns with the major series published in the literature, where these injury mechanisms are closely associated with extensive capsuloligamentous disruption and frequent neurovascular involvement [11,14].

Regarding the distribution of injury patterns, the most frequent Schenck type in our cohort was KD V (40%), followed by KD I and KD IV. This observation is consistent with the findings of Levy et al. [15], who reported a higher incidence of combined injuries affecting cruciate ligaments and collateral structures. Knee dislocation was present in 40% of the cases in our study, within the expected range reported by Stannard et al. (37% to 50%) [14].

From a therapeutic standpoint, anatomical multiligament reconstruction, primarily following the LaPrade technique or its anatomical variants, was the most commonly employed procedure (44%). This technique has demonstrated its ability to accurately restore the anatomy and biomechanics of the posterolateral corner, effectively re-establishing varus and rotational stability with excellent functional outcomes [11,12,14]. In our series, complex reconstructions requiring additional fixation or plating represented 20% of cases, reflecting the high frequency of associated bony injuries and the need for combined surgical strategies in high-energy trauma scenarios [16,17].

Our overall complication rate (20%) was comparable to that reported in the literature (20% to 30%) [14,18]. Postoperative stiffness was the most common complication, often related to prolonged immobilization or the use of temporary external fixation [19]. Avascular necrosis of the medial femoral condyle, although infrequent, represented a clinically significant complication, particularly in the context of extensive reconstructive procedures or local vascular compromise [20]. Re-ruptures of the ACL, progressive chondropathy, and persistent meniscal injuries highlight the difficulty of completely restoring joint biomechanics in knees subjected to high-energy trauma. Notably, all these complications occurred in patients who sustained high-energy traumatic mechanisms, specifically traffic-related accidents, suggesting a potential association between injury severity and postoperative adverse outcomes.

Importantly, no deep infections, compartment syndromes, or postoperative neurovascular complications were observed. This favorable profile may be attributed to early multidisciplinary management, optimized soft-tissue control, and structured rehabilitation protocols [21]. Overall, the outcomes of this series support the use of anatomical multiligament reconstruction, particularly using the LaPrade technique, as an effective and reliable strategy for the management of these complex injuries.

This study has inherent limitations, including its retrospective single-center design and limited sample size (n = 25), which restrict statistical power and generalizability. Furthermore, heterogeneity in injury patterns, surgical techniques, and perioperative management over the 15-year study period may have introduced treatment variability and temporal bias related to the evolution of surgical practices and rehabilitation protocols. Functional outcomes were patient-reported and subjective, as no validated scoring systems were used, limiting reproducibility and objective comparability with other studies.

Despite these limitations, this study provides clinically relevant descriptive data from a tertiary referral center with long-standing experience in the management of multiligament knee injuries. The findings offer valuable insight into injury mechanisms, treatment strategies, and complication profiles, and support the role of anatomical reconstruction within a tailored, protocol-driven approach, particularly in patients sustaining high-energy trauma.

## Conclusions

Multiligament knee injuries are uncommon yet highly complex conditions that predominantly affect young male patients following high-energy trauma, particularly traffic-related accidents. In this retrospective descriptive series, anatomical multiligament reconstruction, most commonly performed using LaPrade-based techniques, was the preferred surgical approach. Patient-reported functional outcomes were satisfactory in 60% of cases and should be interpreted as descriptive and exploratory findings rather than definitive measures of treatment efficacy. The overall complication rate of 20% was comparable to that reported in previous studies, with stiffness, condylar necrosis, and ligament graft failure being the most relevant adverse events. While early multidisciplinary management and protocolized rehabilitation appear to play an important role in postoperative recovery, further studies using validated outcome measures and comparative designs are needed to better define functional outcomes and treatment effectiveness in this patient population.
